# Whole-Genome Sequencing and Comparative Genomic Analysis of Three Clinical Bloodstream Infection Isolates of *Trichosporon austroamericanum*

**DOI:** 10.3390/jof11050401

**Published:** 2025-05-21

**Authors:** Takanori Horiguchi, Takashi Umeyama, Hiroko Tomuro, Amato Otani, Takayuki Shinohara, Masahiro Abe, Shogo Takatsuka, Ken Miyazawa, Minoru Nagi, Yasunori Muraosa, Yasutaka Hoshino, Takashi Sakoh, Hideki Araoka, Naoyuki Uchida, Tomoyoshi Kaneko, Yuko Nagano, Hiroki Tsukada, Taiga Miyazaki, Yoshitsugu Miyazaki

**Affiliations:** 1 Department of Fungal Infection, National Institute of Infectious Diseases, Japan Institute for Health Security, Tokyo 162-8640, Japan or takanori_horiguchi@med.miyazaki-u.ac.jp (T.H.); kusachi@niid.go.jp (H.T.); a-otani@niid.go.jp (A.O.); taka-s@niid.go.jp (T.S.); masa-a@niid.go.jp (M.A.); tktk@niid.go.jp (S.T.); k-miyazawa@niid.go.jp (K.M.); ymuraosa@niid.go.jp (Y.M.); hyasu@niid.go.jp (Y.H.); ym46@niid.go.jp (Y.M.); 2 Division of Respirology, Rheumatology, Infectious Diseases and Neurology, Department of Internal Medicine, Faculty of Medicine, University of Miyazaki, Miyazaki 889-1692, Japan; taiga_miyazaki@med.miyazaki-u.ac.jp; 3 Antimicrobial Resistance Research Center, National Institute of Infectious Diseases, Japan Institute for Health Security, Tokyo 189-0002, Japan; nagi@niid.go.jp; 4 Department of Infectious Diseases, Toranomon Hospital, Tokyo 105-8470, Japan; paramount.saga@gmail.com (T.S.); h-araoka@toranomon.gr.jp (H.A.); 5 Department of Hematology, Toranomon Hospital, Tokyo 105-8470, Japan; nuchida@toranomon.gr.jp; 6 The Jikei University School of Medicine Kashiwa Hospital, Chiba 277-8567, Japan; t_kaneko@jikei.ac.jp (T.K.); y_nagano@jikei.ac.jp (Y.N.); 056773htsukada@jikei.ac.jp (H.T.)

**Keywords:** *Trichosporon austroamericanum*, whole-genome sequencing, comparative genomics, bloodstream infections, zinc cluster transcription factor, pathogenicity, ortholog analysis, *Galleria mellonella*

## Abstract

*Trichosporon austroamericanum* is a recently described yeast species related to *Trichosporon inkin* and exclusively isolated from clinical specimens. However, its genomic features and pathogenic potential remain poorly understood. In this study, we performed whole-genome sequencing on three blood-derived isolates from patients with invasive fungal infections and comparative analyses with 13 related *Trichosporon* species. The three isolates yielded high-quality assemblies of 9–10 scaffolds (~21 Mb), facilitating reliable comparisons. While most species had comparable genome sizes, *Trichosporon ovoides*, *Trichosporon coremiiforme*, and *Cutaneotrichosporon mucoides* displayed large, fragmented genomes, suggestive of polyploidy. ANI analysis and phylogenetic trees based on ANI and single-copy orthologs supported the classification of *T. austroamericanum* as a distinct clade with moderate intraspecific divergence. Using the *Galleria mellonella*, a model for fungal pathogenicity, all *T. austroamericanum* strains reduced larval survival, and NIIDF 0077300 exhibited virulence comparable to *T. asahii* and greater than *T. inkin*. To explore the gene-level differences associated with pathogenicity, we performed ortholog analysis based on single-copy genes. This revealed a unique Zn(II)_2_Cys_6_-type transcription factor gene (OG0010545) present only in NIIDF 0077300 and *T. asahii*. These findings highlight the genomic diversity and infection-associated traits of *T. austroamericanum*, providing a framework for future functional studies.

## 1. Introduction

*Trichosporon* is a genus of basidiomycetous yeasts within the phylum *Basidiomycota*, widely distributed in natural environments, such as soil, water, and decaying organic matter, and is found as a commensal organism in the human microbiota [1,2]. While generally regarded as harmless, several *Trichosporon* species have emerged as opportunistic pathogens capable of causing invasive infections, particularly in immunocompromised individuals [1,2,3,4]. Among these, *Trichosporon inkin* is considered a clinically important species that is commonly associated with superficial infections, although cases of invasive trichosporonosis have also been reported in patients with hematologic malignancies or neutropenia. However, such systemic infections are exceedingly rare [4,5,6,7,8].

*Trichosporon austroamericanum* is a recently described yeast species isolated from clinical specimens and is closely related to *T*. *inkin*, based on sequence analysis of the intergenic spacer 1 (IGS1) region [9]. Standard barcoding regions, such as the Internal Transcribed Spacer and D1/D2, share >99% identity between these two species, making it difficult to reliably distinguish them using conventional markers [9]. In contrast, IGS1 analysis enables reliable species-level differentiation and has proven useful in refining the *Trichosporon* taxonomy. Some isolates previously identified as *T*. *inkin* have been reassigned to *T*. *austroamericanum* [9]. *T*. *austroamericanum* has been isolated exclusively from clinical specimens, including urine and skin, and has been reported in geographically diverse regions, such as China, Brazil, France, Argentina, and India [9]. In Japan, three isolates with IGS1 sequences identical to those of *T*. *austroamericanum* were recently recovered from the blood of patients with bloodstream infections, suggesting that this species may possess a previously unrecognized pathogenic potential.

Despite its isolation in patients with invasive infections and close genetic relatedness to clinically important *Trichosporon* species, genomic insights into *T*. *austroamericanum* are currently lacking, hindering the understanding of its evolutionary relationships and pathogenic potential. Whole-genome sequencing (WGS) has become the cornerstone of modern phylogenetic classification, enabling precise species delineation at the genomic level. Beyond taxonomy, WGS further reveals lineage-specific genomic features that may underlie phenotypic traits such as pathogenicity, drug resistance, and environmental adaptation [10,11]. We selected WGS as our primary approach because conventional barcoding markers such as ITS and D1/D2 are insufficient to resolve the taxonomic ambiguity between *T. austroamericanum* and closely related species. In addition to WGS, we applied genome-wide average nucleotide identity (ANI) analysis and ortholog-based phylogenetics to assess interspecies relatedness from both nucleotide and protein perspectives. Comparative genomics was also employed to explore strain-specific features that may explain clinical adaptation. Together, these approaches enabled us to clarify the taxonomic position of *T. austroamericanum* and gain insight into its genomic features.

## 2. Materials and Methods

### 2.1. Fungal Isolates

All strains used in this study are listed in Table 1.

Three strains of *T*. *austroamericanum* (NIIDF 0077000, NIIDF 0077300, and NIIDF 0079200) were isolated from blood cultures collected between 1 April 2023 and 31 March 2024. All three strains were detected in blood culture specimens obtained from different patients. NIIDF 0077000 and NIIDF 0079200 were isolated at the same hospital, while NIIDF 0077300 was obtained from a hospital located in a different city. Thirteen *Trichosporon*-related strains, excluding *T*. *austroamericanum*, were obtained from the Japan Collection of Microorganisms (JCM), RIKEN BRC.

### 2.2. DNA Extraction and Genome Sequencing

Each strain used in this study was cultured on Potato Dextrose Agar (PDA; Difco™, Becton, Dickinson and Company, Franklin Lakes, NJ, USA) at 30 °C for 48 h. A single colony was aseptically transferred to 10 mL of Yeast Extract Peptone Dextrose (YPD) broth (Difco™, Becton, Dickinson and Company, Franklin Lakes, NJ, USA) and incubated at 30 °C with shaking at 150 rpm for 24 h. After cultivation, cells were centrifuged at 3000× *g* for 10 min, and the resulting pellet was washed once with 1× phosphate-buffered saline (PBS) by centrifugation. The resulting pellet was frozen in liquid nitrogen, ground using a mortar and pestle, and subjected to genomic DNA extraction using the Maxwell^®^ RSC Genomic DNA Kit (Promega, Madison, WI, USA) according to the manufacturer’s protocol. DNA purity (A_260_/A_280_ ratio) was assessed using a Nanodrop spectrophotometer (Thermo Fisher, Waltham, MA, USA), and DNA concentration was quantified using a Quantus™ Fluorometer (Promega, Madison, WI, USA). The extracted DNA was barcoded using the Native Barcoding Kit 24 V14 (Oxford Nanopore Technologies, Didcot, UK) according to the manufacturer’s protocol, and sequencing was performed for 24–72 h on an R10.4.1 flow cell (Oxford Nanopore Technologies, Didcot, UK). Basecalling was conducted using Dorado (v0.7.1), and the processed reads were used for subsequent analyses [12].

### 2.3. Genome Assembly

The analysis was performed using the NIG supercomputer at the ROIS National Institute of Genetics (Mishima, Shizuoka, Japan). Nanopore sequencing data were filtered to remove low-quality reads using Filtlong (v0.2.1) with the following parameters: --keep_percent 95--min_length 1000 [13]. Genome assembly was performed using Flye (v2.9.5) with default parameters, followed by read mapping using Minimap2 (v2.24) [14,15]. Contig polishing and consensus generation were conducted using two iterations of Racon (v1.5.0) and one iteration of Medaka (v1.8.0), with default settings [16,17]. Assembly quality was evaluated using QUAST (v5.0.2), whereas genome completeness was assessed using BUSCO (v5.2.2) and the tremellomycetes_odb10 reference database [18,19].

### 2.4. Gene Prediction and Functional Annotation

Gene prediction and functional annotation were performed using Funannotate (v1.8.17) [20]. A combination of evidence-based and ab initio gene prediction methods was used in this study. The tremellomycetes_odb10 BUSCO database was used to extract conserved gene models, which were then utilized to train the ab initio prediction tools AUGUSTUS (v4.0.0), GlimmerHMM (v3.0.4), and SNAP [21,22,23]. Additionally, self-training gene prediction was performed using GeneMark-ES (v4.72) with parameters optimized for fungal genomes [24]. Functional annotation was conducted using InterProScan (v5.70-102.0), which compares the predicted gene models against the Pfam, PROSITE, and SMART databases to identify conserved domains and assign functional descriptions. Gene Ontology (GO) terms were assigned based on InterProScan GO mapping as well as functional categorization using EggNOG-mapper (v2.1.12) [25,26]. Secondary metabolite biosynthetic gene clusters were predicted using AntiSMASH (v7.0.0) to identify putative non-ribosomal peptide synthetases (NRPSs), polyketide synthases (PKSs), and other secondary metabolite pathways. Signal peptides were predicted using SignalP (v5.0b), whereas Phobius (v1.01) was used to predict transmembrane domains and additional signal peptides, facilitating protein localization analysis [27,28,29].

### 2.5. Phylogenetic Analysis

We used two complementary phylogenetic approaches based on genome-wide nucleotide identity and single-copy orthologous genes (SCOs). Pairwise Average Nucleotide Identity (ANI) values were calculated using FastANI (v1.33) to quantify genome-wide similarity [30]. The resulting output was processed via a custom Python (v3.12.3) script into a CSV-formatted ANI matrix that was used in the two downstream analyses. First, the ANI values were transformed into a distance matrix (1−ANI/100), and a distance-based clustering tree was generated using the neighbor-joining (NJ) method implemented in the Bio.Phylo module of Biopython (v1.81) [31,32]. The resulting tree, exported in Newick format, represented hierarchical relationships based on genomic dissimilarity and was used to visualize the overall patterns of relatedness among the analyzed strains. In parallel, the same ANI matrix was imported into R (v4.3.3) and displayed as a heatmap using the ComplexHeatmap package (v2.18.0) to provide an overview of genome-wide similarity patterns across strains [33]. Protein-level phylogenetic inference was performed using OrthoFinder (v2.5.5) [34]. Orthologous groups (OGs) were inferred from predicted protein sequences based on an all-versus-all DIAMOND (v 2.1.9) search [35]. Multiple sequence alignments were generated for each orthogroup using MAFFT (v7.505), and gene trees were constructed with IQ-TREE2 (v2.4.0) using the -M msa -T iqtree option, which performs maximum likelihood (ML) inference from alignments [36,37]. Based on these gene trees, OrthoFinder automatically identified SCOs shared across all analyzed strains and generated a concatenated alignment for species tree estimation. This alignment was subjected to further ML inference using IQ-TREE2 with the best-fit model selected by ModelFinder (-m MFP) implemented in IQ-TREE version 2.4.0 and 1000 ultrafast bootstrap replicates (-bb 1000) to assess branch support [37,38,39]. The final species tree was exported in Newick format. The resulting tree was visualized using FigTree (v1.4.4) [40].

### 2.6. Ortholog Analysis

Ortholog analysis was performed using OrthoFinder [34]. In this analysis, orthologous groups (OGs) that were shared by at least one strain of *T*. *asahii* and *T*. *austroamericanum* but absent in all other analyzed *Trichosporon* species were extracted. A list of protein-coding gene identifiers (FUN IDs) corresponding to these OGs was generated for relevant strains. Functional annotations of these genes were retrieved from the Funannotate output using a custom script focusing on the presence of domains retrieved from InterProScan results via Funannotate. The corresponding protein sequences were obtained in the FASTA format, and a homology search was conducted against the PHI-base (v4.17) using BLASTP (v2.12.0+) with thresholds of E-value ≤ 1 × 10^−5^, sequence identity ≥ 30%, and query coverage ≥ 50% [41].

### 2.7. Galleria mellonella Infection Model

Each strain was cultured as described above, and the cells were centrifuged at 3000× *g* for 10 min to collect the pellet. The cells were then resuspended in PBS, and cell density was manually determined and adjusted using a disposable hemocytometer. The fungal suspension was prepared at a final concentration of 1 × 10^6^ CFU per larva. Groups of *Galleria melonella* larvae (*n* = 30 per strain per experiment) were inoculated with 10 µL of the suspension in the left proleg using a Hamilton syringe (Trajan Scientific and Medical, Ringwood, Victoria, Australia). After injection, the larvae were placed in Petri dishes and maintained at 37 °C in the dark throughout the observation period [42]. Survival curves of *G*. *mellonella* larvae were constructed using the Kaplan–Meier method based on daily survival monitoring for up to 10 days post-infection. To compare survival distributions between groups, the log-rank test (Mantel–Cox test) was performed. To account for multiple pairwise comparisons among strains and the control group, the Bonferroni correction was applied to the resulting *p*-values. Statistical analyses were conducted using the Python lifelines package (version 0.30.0), and survival curves were visualized using matplotlib (v 3.10.0) [43,44].

## 3. Results

### 3.1. Genome Assembly and Gene Annotation

The genome structure and gene content of *T*. *austroamericanum* strains NIIDF 0077000, NIIDF 0077300, and NIIDF 0079200 were evaluated to assess assembly quality and genomic stability. The genomes were assembled into 9–10 scaffolds, with an average size of 21.04 Mb and an N50 of 3.19–3.88 Mb (Table 2).

Each strain encoded approximately 7427–7574 protein-coding genes and 323–344 tRNA genes. In comparison, there were ten other *Trichosporon* and related strains (excluding *Cutaneotrichosporon mucoides* JCM 9939, *Trichosporon coremiiforme* JCM 2938, and *Trichosporon ovoides* JCM 9940), which showed genome sizes of 18.8–22.3 Mb and 6946–9054 protein-coding genes, indicating similar gene content and size. The three excluded strains displayed much larger genomes (41.3–42.2 Mb), approximately double the gene counts, and elevated tRNA gene numbers (759–930), suggesting possible polyploidy. BUSCO analysis confirmed the high completeness and low duplication of *T*. *austroamericanum* genomes, with Complete BUSCOs ranging from 90.3% to 91.4%, and Duplicated Complete BUSCOs at only 0.1% (Table 3).

These values were comparable to those of other non-polyploid strains. In contrast, the three suspected polyploid strains exhibited markedly high duplication rates (63.9–70.6%). The annotation coverage of *T*. *austroamericanum* was also stable and consistent across the strains: 75.7–76.4% for InterProScan, 83.1–83.6% for eggNOG, and 66.5–66.9% for Pfam. Similar values were observed for other non-polyploid strains (Table 4).

Functional classification using CAZyme (annotated using dbCAN HMMdb release 13.0), MEROPS (v12.5), and Phobius (v1.01) revealed no unusual deviations.

### 3.2. Phylogenetic Analysis

**Figure 1:**
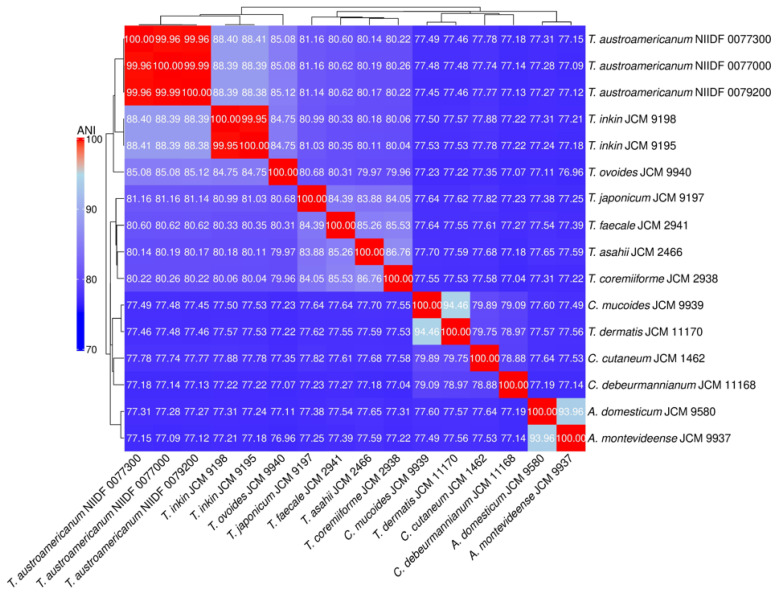
Heatmap and hierarchical clustering of genome-wide ANI among Trichosporonales strains.

**Figure 2:**
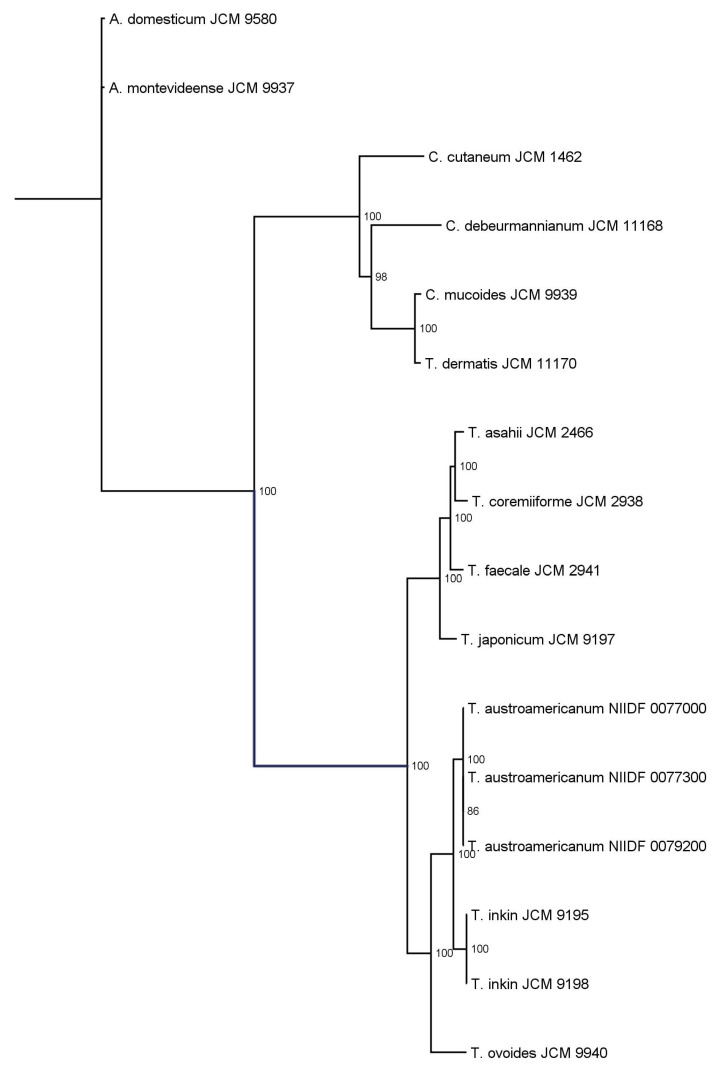
Maximum likelihood phylogenetic tree inferred from single-copy orthologs identified in 16 Trichosporonales strains.

To characterize the genomic distinctiveness of *T*. *austroamericanum*, we conducted phylogenetic analyses using two complementary approaches: whole-genome ANI (Figure 1) and single-copy ortholog (SCO)-based ML phylogeny (Figure 2).

Pairwise Average Nucleotide Identity (ANI) values among the 16 Trichosporonales strains were calculated using FastANI and visualized as a heatmap. Red shading indicates high genomic similarity (higher ANI values), whereas blue shading indicates low similarity. The dendrogram represents hierarchical clustering based on a distance matrix derived from ANI values (1–ANI/100).

Orthologous gene groups were identified using OrthoFinder, and a set of single-copy ortholog genes conserved across all strains was extracted. Multiple sequence alignments were performed using MAFFT, concatenated into a supermatrix, and subjected to phylogenetic inference using the IQ-TREE2. The best-fit model was selected using ModelFinder, and branch support was assessed using 1000 ultrafast bootstrap replicates. Ultrafast bootstrap support (UFBoot) values are shown at internal nodes as percentages, with only values ≥70% displayed. The tree resolved the genera *Apiotrichum*, *Cutaneotrichosporon*, and *Trichosporon* into distinct clades. Notably, the node separating *Apiotrichum domesticum* and *A*. *montevideense* lacked bootstrap support, indicating a low statistical confidence in their divergence.

ANI analysis provided a measure of genome-wide nucleotide similarity, whereas SCO-based phylogeny inferred evolutionary relationships based on conserved protein-coding genes. The three *T*. *austroamericanum* strains exhibited extremely high similarities (ANI = 99.96–99.99%), indicating that they constituted a single genomic lineage. ANI values between *T*. *austroamericanum* and *T*. *inkin* ranged from 88.38% to 88.44%, well below the 95% species threshold, supporting their separation. Lower ANI values (~84.68–85.15%) were observed for *T*. *ovoides*, *T*. *japonicum*, and *T*. *faecale*, and even lower ANI values (<78%) for *Cutaneotrichosporon* and *Apiotrichum* species, reflecting increasing evolutionary distance. The SCO-based ML phylogeny similarly recovered *T*. *austroamericanum* as a strongly supported monophyletic clade (ultrafast bootstrap support [UFBoot] = 100), indicating a highly robust grouping. Within this group, strains 0077000 and 0077300 formed a sister pair with moderate support (UFBoot = 86.0), suggesting early-stage intraspecific divergence. The tree clearly separated the genera *Apiotrichum*, *Cutaneotrichosporon*, and *Trichosporon*, which was consistent with the current taxonomy. However, the node separating *Apiotrichum domesticum* and *Apiotrichum montevideense* lacks statistical support, leaving the relationship unresolved. Together, the ANI and ML trees provided complementary perspectives on both species- and strain-level relationships within the Trichosporonales and reinforced the distinctiveness of *T*. *austroamericanum*.

### 3.3. Virulence Assessment Using Galleria mellonella Infection Model

**Figure 3:**
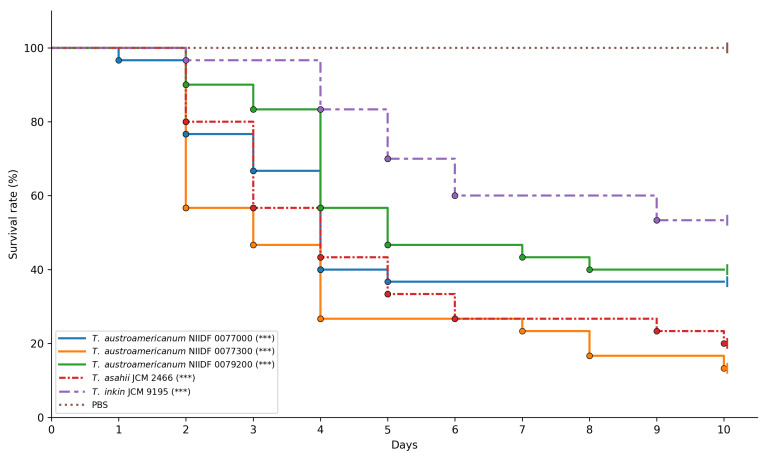
*Galleria mellonella* survival after fungal infection. Kaplan–Meier survival curves of *Galleria mellonella* larvae (*n* = 30 per strain) infected with *Trichosporon* isolates at an inoculum of 1 × 10^6^ CFU per larva. Larvae injected with PBS served as the control group. Asterisks indicate statistically significant differences compared to the PBS group (log-rank test with Bonferroni correction): *** *p* < 0.001. Censored individuals (alive on day 10) are indicated by the vertical tick marks on the curves.

To assess strain-specific differences in virulence and provide a phenotypic context for orthologous gene analysis, we used the *G*. *mellonella* infection model [41]. Three bloodstream-derived isolates of *T*. *austroamericanum* (NIIDF 0077000, 0077300, and 0079200) were compared with *T*. *asahii* JCM 2466, a well-documented cause of invasive infections, and *T*. *inkin* JCM 9195, a phylogenetically related species generally associated with superficial infections. At an inoculum of 1 × 10^6^ CFU per larva, all strains caused markedly increased larval mortality relative to baseline survival (Figure 3).

Pairwise comparisons revealed that *T*. *austroamericanum* NIIDF 0077300 and *T*. *asahii* JCM 2466 exhibited significantly higher virulence than *T*. *inkin* JCM 9195 (adjusted *p* = 0.0005 and 0.0154, respectively) (Table 5).

In contrast, NIIDF 0077000 and NIIDF 0079200 showed no statistically significant differences from *T*. *inkin* (adjusted *p* = 0.5687 and 1.0000, respectively). No significant differences were observed among the *T*. *austroamericanum* strains or between any of them and *T*. *asahii*. Among the strains tested, NIIDF 0077300 and *T. asahii* JCM 2466 demonstrated relatively high virulence, with larval survival below 30% by day 10 (Figure 3). In contrast, *T*. *inkin* JCM 9195 and the remaining *T*. *austroamericanum* strains (NIIDF 0077000 and 0079200) exhibited moderate virulence under the same conditions.

### 3.4. Ortholog Analysis

As shown in the survival curve (Figure 3), all three strains of *T*. *austroamericanum* showed higher virulence in the *G*. *mellonella* infection model than in the PBS control. Notably, the virulence profiles of these strains were comparable to those of *T*. *asahii* JCM 2466, a well-characterized pathogenic species. Based on these phenotypic similarities, we selected three strains of *T*. *austroamericanum* and *T*. *asahii* for comparative ortholog analysis to examine the shared gene content that might be related to their observed virulence profiles. This approach was designed to link phenotypic observations with genomic features identified through comparative analysis. Using OrthoFinder, 15432 OGs were identified across 16 *Trichosporon*-related strains. Among them, we extracted OGs that were common to the two species but absent from all other *Trichosporon* species. Consequently, a single OG (OG0010545) was identified. OG0010545 was found only in *T. asahii* JCM 2466 and *T. austroamericanum* strain NIIDF 0077300 and was absent in the other two *T. austroamericanum* strains (NIIDF 0077000 and NIIDF 0079200) as well as in *T. inkin* JCM 9195. The genes were annotated by Funannotate as FUN_006829-T1 and FUN_007303-T1, respectively. Although both genes were labeled as “hypothetical protein” with no clearly assigned function, they possessed conserved domains associated with Zn(II)_2_Cys_6_ fungal-type DNA-binding transcription factors (IPR001138, IPR036864). GO annotations for both genes included terms such as zinc ion binding (GO:0008270), RNA polymerase II-specific DNA-binding transcription factor activity (GO:0000981), and regulation of DNA-templated transcription (GO:0006355). EggNOG analysis further classified both genes into the same orthologous group (ENOG503P0AT), indicating evolutionary conservation. A PHI-based search yielded no matches with the annotated virulence functions. These results highlight that OG0010545 is a conserved orthologous group uniquely shared between *T*. *asahii* and *T*. *austroamericanum* strain NIIDF 0077300, with features consistent with those of fungal transcription factors.

## 4. Discussion

In this study, we explored the genomic features and infection model responses of *T*. *austroamericanum*, a yeast species recently isolated from blood culture. Taken together, the results presented in Table 2, Table 3 and Table 4—covering assembly metrics, genome completeness, and functional annotation—demonstrate that all the analyzed strains possess high-quality, contiguous, and stable genomes with a conserved functional composition, supporting their suitability for comparative and functional genomic studies. WGS and comparative analyses were conducted to characterize the genome structure and clarify its phylogenetic position within the genus *Trichosporon*. To this end, we employed two complementary phylogenomic approaches: ANI (Figure 1), which quantifies genome-wide sequence similarity at the nucleotide level, and ML phylogeny based on SCOs (Figure 2), which reconstructs evolutionary relationships from conserved protein-coding genes. In the ANI heatmap (Figure 1), the three *T*. *austroamericanum* strains clustered tightly, exhibiting pairwise ANI values of 99.96–99.99%, consistent with extremely high genomic similarity. These strains formed a visually distinct block, clearly separated from other *Trichosporon* species, most notably from *T*. *inkin*, with ANI values ranging from 88.38% to 88.44%. This value fell well below the 95% threshold commonly used for species delineation, supporting the interpretation that *T*. *austroamericanum* is genomically distinct. A gradual decline in ANI values with increasing evolutionary distance was also observed, which aligned with the known taxonomic relationships. Similarly, the SCO-based ML tree (Figure 2) identified *T*. *austroamericanum* as a strongly supported monophyletic clade (UFBoot = 100). Within this clade, strains NIIDF 0077000 and 0077300 formed a moderately supported sister group (UFBoot = 86.0), suggesting early-stage divergence despite their nearly identical ANI values. This internal structuring within *T*. *austroamericanum* may reflect incipient genomic diversification, potentially resulting in strain-specific adaptation or functional divergence. The overall tree topology further classified the genera *Apiotrichum*, *Cutaneotrichosporon*, and *Trichosporon* into well-supported clades. However, the node separating *A*. *domesticum* and *A*. *montevideense* lacked bootstrap support, indicating that their relationship remains unresolved at the current resolution. Taken together, the ANI-based similarity landscape and protein-level ML phylogeny offer complementary perspectives on evolutionary divergence, highlighting both inter-species boundaries and emerging intraspecific variation. These findings reinforce the genomic distinctiveness of *T*. *austroamericanum* and underscore its status as a separate lineage that is closely related to *T*. *inkin*.

To assess strain-specific differences in virulence and provide a phenotypic context for orthologous gene analysis, we used the *G*. *mellonella* infection model. We compared the virulence of three clinical isolates of *T*. *austroamericanum*—strains NIIDF 0077000, 0077300, and 0079200—with *T*. *asahii* JCM 2466, a well-documented cause of invasive infections, and *T*. *inkin* JCM 9195, a phylogenetically related species typically associated with superficial infections [42]. At an inoculum of 1 × 10^6^ CFU per larva, all fungal strains tested induced significantly increased *G*. *mellonella* mortality compared to the PBS control (log-rank test, *** *p* < 0.001; Figure 3). Among the *T*. *austroamericanum* strains, NIIDF 0077300 exhibited a virulence profile closely resembling that of *T*. *asahii* and significantly different from that of *T*. *inkin* (adjusted *p* = 0.0005). However, no statistically significant differences were observed between NIIDF 0077300 and the other *T*. *austroamericanum* strains (NIIDF 0077000 and 0079200) or between these strains and *T*. *inkin* (adjusted *p* = 0.5687 and 1.0000, respectively). Additionally, there were no significant differences among the *T*. *austroamericanum* strains or between any of them and *T*. *asahii*. These findings demonstrate that all tested *T. austroamericanum* strains are capable of inducing lethal infection in the *Galleria mellonella* model, while also exhibiting strain-dependent variability in virulence. Furthermore, the observed mortality profiles suggest that *T. austroamericanum* exhibits strain-dependent virulence potential, with NIIDF 0077300 displaying pathogenicity comparable to that of *T. asahii*, whereas the other two strains showed levels of virulence similar to *T. inkin*. This is consistent with previous work by Mariné et al. [42], who reported that *T. asahii* was significantly more virulent than *T. inkin* in the *G. mellonella* model, as measured by survival curves and time to death. Our findings parallel this pattern, suggesting that *T. austroamericanum* may encompass strains with divergent virulence potential comparable to either species. Although the genetic basis of this phenotypic diversity remains to be elucidated, it may partly reflect underlying genomic differences. These observations highlight the importance of integrating phenotypic evidence with ortholog-based comparative analyses in future studies.

In ortholog analysis, OG0010545 was identified as an orthologous group shared between *T*. *asahii* and the most virulent *T*. *austroamericanum* strain NIIDF 0077300. It was absent in all the other strains examined. All genes within this ortholog group encode proteins with conserved Zn(II)_2_Cys_6_-type DNA-binding domains (IPR001138, IPR036864), suggesting that they likely encode fungal-specific transcription factors of the Zn(II)_2_Cys_6_ family. Zn(II)_2_Cys_6_-type transcription factors (zinc cluster proteins) are fungal-specific regulatory proteins known to perform diverse functions depending on the species, including metabolic regulation, drug resistance, ergosterol biosynthesis, and control of morphological transitions [45]. Structurally, Zn(II)_2_Cys_6_ transcription factors possess a conserved N-terminal DNA-binding domain (DBD) characterized by six cysteines arranged in the consensus Cys-X_2_-Cys-X_6_-Cys-X_5–12_-Cys-X_2_-Cys-X_6–8_-Cys motif. This motif coordinates two zinc ions, stabilizing the domain for specific DNA binding, typically to CGG triplet motifs. DBD includes a zinc finger, linker, and dimerization region that facilitates DNA recognition and protein interactions. Additional regulatory and activation domains enable transcriptional control through cofactors and chromatin-modifying complexes. This modular architecture allows for the precise condition-dependent regulation of target genes in fungi [45]. The functional relevance of this transcription factor family in pathogenicity is particularly well studied in *Candida albicans*, a dimorphic fungus capable of switching between yeast and hyphal forms, a morphological transition closely linked to virulence and tissue invasion [46,47,48]. Hyphal development facilitates tissue invasion, endothelial translocation, and immune evasion during systemic infection. Among the 77 candidate transcription factors with Zn(II)_2_Cys_6_ DNA-binding motifs identified in *C*. *albicans*, several, including CZF1, FGR17, and FGR27, regulate this yeast-to-hypha transition and are directly implicated in its pathogenic potential [45]. Given the structural and functional conservation of this transcription factor family, it is conceivable that the Zn(II)_2_Cys_6_-type transcription factor encoded by OG0010545 may regulate downstream genes involved in processes relevant to systemic infection, such as host tissue invasion, endothelial translocation, and immune evasion. Although based solely on in silico predictions, the identification of OG0010545, which encodes a Zn(II)_2_Cys_6_-type transcription factor, in both *T*. *asahii* and the most virulent *T*. *austroamericanum* strain suggests a potential role in infection-related processes. Its presence in these pathogenic strains represents only a single example but raises the possibility that similar regulatory mechanisms contribute to traits associated with persistence or dissemination within the host. Its strain-specific presence in *T*. *austroamericanum* suggests intraspecific genomic variation that may underlie phenotypic differences in host interactions. The presence of this ortholog in phylogenetically distinct, yet pathogenic strains may reflect shared selective pressures associated with host adaptation, potentially involving convergent evolution or horizontal gene retention under similar environmental constraints. This is consistent with the observed phylogenetic substructure and variability among the three isolates. However, the absence of OG0010545 in the other two *T*. *austroamericanum* strains highlights the need for further investigation. Future studies incorporating RNA-Seq under infection-relevant conditions and gene disruption analyses are essential to clarify its potential functional role. It is also likely that additional genomic elements contribute to strain-level variations, warranting further exploration.

This study had several limitations that warrant consideration. First, our conclusions regarding species-specific orthologs were based solely on in silico predictions, including domain architecture and sequence homology, without supporting the transcriptomic data. Although such predictions provide a useful foundation for hypothesis generation, they do not directly confirm the gene function or expression under biologically relevant conditions. To address this limitation, future studies should incorporate transcriptomic analyses, such as RNA-seq, to assess the expression of candidate orthologs under infection-relevant conditions and functional assays, such as gene disruption or complementation, to experimentally validate their biological roles. Second, this study did not use vertebrate infection models. Invertebrate models such as *G*. *mellonella* are widely used for screening fungal pathogenicity and virulence factors, offering practical advantages, including low cost, ease of handling, and exemption from ethical approval [49,50]. However, these models do not fully recapitulate the mammalian immune responses. Although valuable for initial pathogenicity assessment, validation in vertebrate systems, such as murine models, will be important to contextualize the findings within a mammalian host environment. Third, our genome assemblies were generated using long-read sequencing only, without incorporating short-read correction or hybrid assembly approaches. While the overall assembly quality was high for most strains (9–10 scaffolds), three species showed highly fragmented assemblies. This could reflect either biological features such as polyploidy or technical issues such as degraded or sheared input DNA. Moreover, the absence of short-read polishing may allow residual base-level errors to persist. Future studies should consider hybrid assembly methods, combining long- and short-read sequencing to improve assembly accuracy and completeness, particularly for structurally complex or repetitive regions.

Phylogenetic analyses based on ANI and SCOs indicated that *T*. *austroamericanum* constitutes a distinct lineage within Trichosporonales, despite its close relationship with *T*. *inkin*. The identification of a strain-specific ortholog opens avenues for future research on intraspecific variation in host interaction traits.

## Figures and Tables

**Table 1:** Strains used in this study.

Strains	Strain ID	Source
*Trichosporon austroamericanum*	NIIDF 0077000	human blood
*Trichosporon austroamericanum*	NIIDF 0077300	human blood
*Trichosporon austroamericanum*	NIIDF 0079200	human blood
*Trichosporon inkin*	JCM 9195	human skin
*Trichosporon inkin*	JCM 9198	surgical wound
*Trichosporon ovoides*	JCM 9940	scalp
*Trichosporon asahii*	JCM 2466	human skin
*Trichosporon coremiiforme*	JCM 2938	lesion on farmer head
*Trichosporon dermatis*	JCM 11170	human skin
*Trichosporon faecale*	JCM 2941	human feces
*Trichosporon japonicum*	JCM 9197	unknown
*Apiotrichum domesticum*	JCM 9580	damp and rotten wooden sideboard
*Apiotrichum montevideense*	JCM 9937	water purification tank
*Cutaneotrichosporon cutaneum*	JCM 1462	unknown
*Cutaneotrichosporon debeurmannianum*	JCM 11168	human bronchial secretion
*Cutaneotrichosporon mucoides*	JCM 9939	case of meningitis

**Table 2:** Genome assembly statistics and repeat content in *Trichosporon* and related species sequenced in this study based on QUAST analysis.

Strains	Scaffolds	Total Length (Mb)	Largest Scaffold (Mb)	GC (%)	N50 (Mb)	Repeat Sequences (%)
*T*. *austroamericanum* NIIDF 0077000	9	21.02	3.93	50.03	3.19	19.37
*T*. *austroamericanum* NIIDF 0077300	10	21.05	5.69	50.01	3.88	19.39
*T*. *austroamericanum* NIIDF 0079200	9	21.06	3.92	50.04	3.41	19.32
*T*. *inkin* JCM 9195	36	20.36	3.66	50.01	2.71	18.62
*T*. *inkin* JCM 9198	36	20.44	4.50	50.02	2.74	18.68
*T*. *ovoides* JCM 9940	55	40.40	3.19	50.01	1.87	19.89
*T*. *asahii* JCM 2466	40	24.82	4.04	49.99	2.29	20.28
*T*. *coremiiforme* JCM 2938	153	43.00	1.53	50.00	0.91	20.13
*T*. *dermatis* JCM 11170	24	23.42	3.89	49.90	2.78	20.01
*T*. *faecale* JCM 2941	58	24.81	2.90	50.06	1.37	19.84
*T*. *japonicum* JCM 9197	28	23.55	4.60	50.07	3.28	18.57
*A*. *domesticum* JCM 9580	20	24.65	7.92	49.98	3.53	20.77
*A*. *montevideense* JCM 9937	24	25.18	3.76	49.96	2.17	20.87
*C*. *cutaneum* JCM 1462	122	24.22	1.65	50.06	0.37	18.73
*C*. *debeurmannianum* JCM 11168	28	19.15	3.80	49.92	2.97	20.34
*C*. *mucoides* JCM 9939	36	42.75	4.21	49.96	3.35	19.90

**Table 3:** BUSCO-based assessment of genome completeness, duplication, and gene content integrity in *Trichosporon* related species.

Strains	Complete (%)	Single-Copy (%)	Duplicated (%)	Fragmented (%)	Missing (%)
*T*. *austroamericanum* NIIDF 0077000	91.4	91.3	0.1	2.8	5.8
*T*. *austroamericanum* NIIDF 0077300	91.3	91.2	0.1	2.9	5.8
*T*. *austroamericanum* NIIDF 0079200	90.3	90.2	0.1	2.8	6.5
*T*. *inkin* JCM 9195	88.3	87.9	0.4	4.1	7.6
*T*. *inkin* JCM 9198	88.3	87.9	0.4	4.1	7.6
*T*. *ovoides* JCM 9940	95.3	24.7	70.6	1.1	3.6
*T*. *asahii* JCM 2466	88.4	87.7	0.7	4.8	6.8
*T*. *coremiiforme* JCM 2938	91.0	23.4	67.6	3.4	5.6
*T*. *dermatis* JCM 11170	92.1	91.2	0.9	2.8	5.1
*T*. *faecale* JCM 2941	88.3	87.7	0.6	4.9	6.8
*T*. *japonicum* JCM 9197	88.2	87.7	0.5	4.8	7.0
*A*. *domesticum* JCM 9580	89.0	88.2	0.8	2.7	8.3
*A*. *montevideense* JCM 9937	88.7	88.0	0.7	2.8	8.5
*C*. *cutaneum* JCM 1462	93.2	92.5	0.7	1.7	5.1
*C*. *debeurmannianum* JCM 11168	91.0	90.4	0.6	2.9	6.1
*C*. *mucoides* JCM 9939	93.9	30.0	63.9	1.9	4.2

**Table 4:** Number and proportion of functionally annotated genes in *Trichosporon* related species.

Strains	Genes	InterProScan (%)	eggNOG (%)	Pfam (%)	CAZyme (%)	MEROPS (%)	Phobius (%)
*T*. *austroamericanum* NIIDF 0077000	7427	5674 (76.4%)	6206 (83.6%)	4974 (67.0%)	185 (2.5%)	232 (3.1%)	517 (7.0%)
*T*. *austroamericanum* NIIDF 0077300	7442	5653 (76.0%)	6202 (83.3%)	4959 (66.6%)	185 (2.5%)	232 (3.1%)	511 (6.9%)
*T*. *austroamericanum* NIIDF 0079200	7574	5736 (75.7%)	6291 (83.1%)	5037 (66.5%)	187 (2.5%)	231 (3.0%)	528 (7.0%)
*T*. *inkin* JCM 9195	7325	5715 (78.0%)	6267 (85.6%)	5031 (68.7%)	184 (2.5%)	230 (3.1%)	494 (6.7%)
*T*. *inkin* JCM 9198	7178	5612 (78.2%)	6149 (85.7%)	4913 (68.4%)	182 (2.5%)	228 (3.2%)	485 (6.8%)
*T*. *ovoides* JCM 9940	14321	10864 (75.9%)	11193 (78.2%)	9494 (66.3%)	348 (2.4%)	440 (3.1%)	943 (6.6%)
*T*. *asahii* JCM 2466	8530	5932 (69.5%)	6530 (76.6%)	5143 (60.3%)	194 (2.3%)	245 (2.9%)	639 (7.5%)
*T*. *coremiiforme* JCM 2938	15235	10972 (72.0%)	12053 (79.1%)	9533 (62.6%)	350 (2.3%)	459 (3.0%)	1063 (7.0%)
*T*. *dermatis* JCM 11170	8285	6246 (75.4%)	6816 (82.3%)	5469 (66.0%)	233 (2.8%)	270 (3.3%)	540 (6.5%)
*T*. *faecale* JCM 2941	8774	5950 (67.8%)	6539 (74.5%)	5147 (58.7%)	191 (2.2%)	242 (2.8%)	681 (7.8%)
*T*. *japonicum* JCM 9197	8199	5874 (71.6%)	6405 (78.1%)	5096 (62.2%)	196 (2.4%)	248 (3.0%)	652 (8.0%)
*A*. *domesticum* JCM 9580	7975	5871 (73.6%)	6430 (80.6%)	5148 (64.6%)	208 (2.6%)	244 (3.1%)	613 (7.7%)
*A*. *montevideense* JCM 9937	14315	10887 (76.1%)	11874 (82.9%)	9560 (66.8%)	403 (2.8%)	466 (3.3%)	959 (6.7%)
*C*. *cutaneum* JCM 1462	8982	6315 (70.3%)	6903 (76.9%)	5524 (61.5%)	202 (2.2%)	296 (3.3%)	597 (6.6%)
*C*. *debeurmannianum* JCM 11168	6946	5553 (79.9%)	6069 (87.4%)	4897 (70.5%)	171 (2.5%)	233 (3.4%)	380 (5.5%)
*C*. *mucoides* JCM 9939	14315	10,887 (76.1%)	11,874 (82.9%)	9560 (66.8%)	403 (2.8%)	466 (3.3%)	959 (6.7%)

**Table 5:** Pairwise comparison of virulence among *Trichosporon* strains based on *Galleria mellonella* survival assay. Pairwise comparisons of *Galleria mellonella* survival curves were conducted using the log-rank (Mantel–Cox) test. Adjusted *p*-values were obtained by applying the Bonferroni correction for multiple tests.

Comparison	Adjusted *p*-Value
*T*. *asahii* JCM 2466 vs. *T*. *austroamericanum* NIIDF 0077000	1.0000
*T*. *asahii* JCM 2466 vs. *T*. *austroamericanum* NIIDF 0077300	1.0000
*T*. *asahii* JCM 2466 vs. *T*. *austroamericanum* NIIDF 0079200	0.9312
*T*. *asahii* JCM 2466 vs. *T*. *inkin* JCM 9195	0.0154
*T*. *inkin* JCM 9195 vs. *T*. *austroamericanum* NIIDF 0077000	0.5687
*T*. *inkin* JCM 9195 vs. *T*. *austroamericanum* NIIDF 0077300	0.0005
*T*. *inkin* JCM 9195 vs. *T*. *austroamericanum* NIIDF 0079200	1.0000
*T*. *austroamericanum* NIIDF 0077000 vs. *T*. *austroamericanum* NIIDF 0077300	0.7089
*T*. *austroamericanum* NIIDF 0077000 vs. *T*. *austroamericanum* NIIDF 0079200	1.0000
*T*. *austroamericanum* NIIDF 0077300 vs. *T*. *austroamericanum* NIIDF 0079200	0.0707

## Data Availability

The generated dataset for this study can be found in the NCBI database under the BioProject accession number PRJNB20459. The data are currently under embargo and will be made publicly available upon acceptance of the manuscript.
